# Reduced Performance Due to Adenoviral Gizzard Erosion in 16-Day-Old Commercial Broiler Chickens in Iran, Confirmed Experimentally

**DOI:** 10.3389/fvets.2021.635186

**Published:** 2021-02-01

**Authors:** Amin Mirzazadeh, Beatrice Grafl, Mohammad Abbasnia, Sobhan Emadi-Jamali, Bahman Abdi-Hachesoo, Anna Schachner, Michael Hess

**Affiliations:** ^1^Department of Clinical Sciences, School of Veterinary Medicine, Shiraz University, Shiraz, Iran; ^2^Clinic for Poultry and Fish Medicine, Department for Farm Animals and Veterinary Public Health, University of Veterinary Medicine Vienna, Vienna, Austria; ^3^Christian Doppler Laboratory for Innovative Poultry Vaccines (IPOV), University of Veterinary Medicine Vienna, Vienna, Austria

**Keywords:** fowl adenovirus serotype 1, gizzard erosion, broilers, natural outbreak, growth retardation, experimental reproduction

## Abstract

Adenoviral gizzard erosion (AGE) in broilers is an emerging infectious disease with negative impact on flock productivity. Despite of known primary etiological role of fowl adenovirus serotype 1 (FAdV-1) in AGE, there are a limited number of field reports worldwide, possibly because the disease is less noticeable and clinically difficult to assess. The present study documents an outbreak of AGE in 16-day-old broiler chickens on a farm in the north of Iran and the reproduction of the disease in an experimental setting. In the field, a sudden onset of mortality was noticed in affected broilers resulting in 6% total mortality and decreased weight gain leading to approximately 1-week delay to reach the target slaughter weight. Necropsy findings in dead broilers revealed black colored content in crop, proventriculus and gizzard together with severe gizzard erosions characterized by multiple black-brown areas of variable size in the koilin layer and mucosal inflammation. Microscopic examination revealed necrotizing ventriculitis marked with severe dissociation of koilin layer and degeneration of glandular epithelium with infiltration of mononuclear inflammatory cells. FAdV-1 was isolated from affected gizzards. Phylogenetic analysis of the hexon loop-1 (L1) sequence of the isolated virus showed 100% identity with pathogenic FAdV-1 strains previously reported from broiler chickens with AGE. Subsequently, an *in vivo* study infecting day-old commercial layer chickens with the field isolate demonstrated characteristic lesions and histopathological changes of AGE together with decreased weight gain in the infected birds. For the first time, the progress of a natural outbreak of AGE in Iran is described and experimental reproduction of the disease is demonstrated. The findings highlight the economic impact of the disease for regional poultry production due to mortality and impaired weight gain of the affected broilers.

## Introduction

Historically, gizzard erosions have been attributed mostly to certain non-infectious nutritional factors, for example, vitamin deficiencies, biogenic amines in fish meal, and mycotoxins (1). An involvement of fowl adenovirus (FAdV) in the etiology of gizzard erosions was first described by Tanimura et al. (2) based on the detection of characteristic intranuclear inclusion bodies in affected gizzard mucosa cells. Thereafter, fowl adenovirus serotype 1 (FAdV-1) was isolated and distinctively characterized from condemned gizzards in slaughterhouses in Japan (3).

In many cases, adenoviral gizzard erosion (AGE) affected birds exhibited no apparent clinical signs and were detected only during slaughterhouse inspections (3–6). Natural outbreaks in broilers between 9 and 36 days of age from Japan (7), Poland (8), Germany (9, 10) and Belgium (11) were noticed due to clinical signs of depression, uneven growth and/or mortality. However, detailed data of the economic impact was documented only once by Grafl et al. (9), reporting significantly decreased weekly weight gain, decreased end weight and increased total mortality of AGE affected flocks in comparison to healthy broilers. Recently, several reports described the disease also in pullets and layers characterized by a negative impact on birds' health and production (12–14).

In different studies FAdV-1, determined as the common causative agent from the majority of AGE outbreaks, was confirmed capable of inducing the disease in specific-pathogen-free (SPF) White Leghorn chickens as well as commercial and SPF broilers in experimental settings, reviewed by Schachner et al. (15). Both vertical and horizontal routes of transmission have been described for the disease (9, 16).

Recently, we reported AGE in broiler chickens detected during a slaughterhouse inspection in Iran without clinical data (17). The present study describes clinical, virological and histopathological investigations of the first natural outbreak of AGE in Iran noticed in 16-day-old broilers characterized by increased mortality and significant growth retardation, altogether helpful to emphasize clinical consequences and economic impact of the disease. Furthermore, clinical signs, characteristic gross lesions, and histopathologic changes of AGE are demonstrated after experimental infection of day-old commercial layer chickens with the field isolate.

## Materials and Methods

### Case History

In January 2019, increased mortality (0.16% per day) was noticed in a commercial broiler farm of 24,800 chickens (Ross 308) with 2 houses (each divided to two barns with a service room in the middle), located in Mazandaran province, Iran. Affected chickens died without premonitory symptoms, starting from day 16 onwards for a period of approximately 2 weeks. Concurrently, estimated 5–10% of the birds within the flock, depending on the barn, were clinically depressed and inappetent, resulting in poor weight gain and decreased flock uniformity. Routine necropsy was performed on affected birds of the different barns and gross pathological lesions were documented. During *post-mortem* examination, eight gizzards were collected for histopathological and virological investigation and stored accordingly in 10% buffered formalin at room temperature or frozen at −20°C, respectively.

### Production Parameters

Production records, including total mortality and average harvest weight, together with the bird condemnation rate at slaughter were recorded for the affected farm. Additionally, in order to compare with regional production performance values, corresponding data from 10 to 22 healthy broiler flocks, depending on the parameter, collected during the same time period were analyzed.

### Histopathology

After fixation in 10% buffered formalin, gizzard samples were embedded in paraffin, sectioned at 3 μm using a Microm HM 360 microtome (Microm Laborgeräte GmbH, Walldorf, Germany) and stained with hematoxylin and eosin (H&E). *In situ* hybridization (ISH) for the detection of FAdV-1 DNA in the gizzard sections was carried out according to a protocol described previously by Liebhart et al. (18) using a species specific DNA probe (5′-CGGGGTCGCAGCAGCTGCAGCTCGCGAGCGGAGAACTCG-3′) based on the FAdV-1 long fiber gene.

### Cell Culture and Virus Isolation

Frozen gizzard samples were thawed and homogenized in phosphate buffered saline (20% wt/vol) containing 1 mg/ml streptomycin and 100,000 IU/ml penicillin using a T 25 digital Ultra-Turrax® (IKA, Staufen, Germany). After three freeze-thaw cycles, tissue homogenates were clarified by centrifugation at 3,000 × g for 15 min. The supernatant was filter sterilized using 0.2 μm syringe filters (VWR, Vienna, Austria) and 500 μl of the sample material was inoculated onto nearly confluent chicken embryo liver (CEL) cells prepared from 14-day-old SPF chicken embryos (VALO Biomedia GmbH, Osterholz-Scharmbeck, Germany) according to a protocol of Schat and Sellers (19) in tissue culture flasks (TC Flask T25; Sarstedt, Nümbrecht, Germany). The cells were incubated at 37.8°C in 5% CO_2_ for 5 days or until a cytopathic effect (CPE) was observed. A sample was considered negative when no CPE was noticed after three passages.

### DNA Extraction, Polymerase Chain Reaction (PCR), Phylogenetic and Sequence Analysis

Viral DNA was extracted from 200 μl of clarified supernatant from cell cultures showing CPE with the DNeasy Blood & Tissue Kit (Qiagen, Vienna, Austria). In order to detect and type FAdVs, extracted DNA was subjected to a conventional PCR, amplifying the loop-1 (L1) region of the hexon gene, followed by sequencing of the PCR product (20). Additionally, samples were investigated using specific primer sets based upon the FAdV-1 long and short fiber genes described previously by Marek et al. (21). Sequencing services were provided by LGC Genomics GmbH (Berlin, Germany). Assembly and nucleotide sequence alignments were conducted with Lasergene package (DNASTAR Inc., Madison, WI, USA). Sequences were aligned using CLUSTAL W method and phylogenetic tree construction was performed with MegAlign software applying 1,000 bootstrap trials. Analysis was carried out by comparing the obtained nucleotide sequences with hexon L1 and fiber sequences available from GenBank database. The nucleotide sequences for partial hexon, long, and short fiber genes obtained in this study have been submitted to GenBank under accession numbers MN165288, MN165289 and MN165290, respectively.

### Pathogenicity of the Field Isolate in Day-Old Commercial Pullets

The mean tissue culture infective dose (TCID_50_) of FAdV-1 strain, IRMGH019, isolated from broilers in the field was determined in CEL cells using the method described by Reed and Muench (22). Commercial layer eggs were obtained from breeders maintained in remote and isolated facilities of the School of Veterinary Medicine, Shiraz University, Shiraz, Iran. Hatched birds were individually marked with a numbered tag applied subcutaneously, weighed and randomly divided into two groups. Group 1 comprised 24 chicks, of which 16 were orally inoculated with 10^7^ TCID_50_/0.1 ml of field isolate at the first day of life while the remaining eight chicks were left uninoculated as in-pen contact birds. Group 2, comprised of six birds, served as negative control group and birds were administered the same amount of sterile PBS as used in group 1 orally. Each group was housed in an individual isolated room on wire floor under negative pressure with controlled ventilation, temperature and light conditions. Water and feed were provided *ad libitum* throughout the trial. In the course of the experiment chickens were monitored for clinical signs and weighted at the following sampling days to investigate growth retardation; at day 7 and 10 post infection (DPI), one control bird, two in-pen contacts, and five infected birds were necropsied after cervical dislocation and *post-mortem* examinations were performed. During necropsy, gross lesions were documented and changes in the koilin layer of gizzards were scored according to Nakamura et al. (23) before samples were collected for histopathological investigation. At 14 DPI, all remaining birds were killed and *post-mortem* examinations were performed accordingly. Gizzard samples were stained using H&E as described above.

### Statistical Analysis

The average body weight of the birds in group 1 (orally infected together with in-pen contact birds) and group 2 (negative control) at 0, 7, 10, and 14 DPI was analyzed by the student *t*-test. Statistical differences with *P* < 0.05 were considered to be significant. Analysis was performed with the statistical software package SPSS Version 25 (IBM SPSS Statistics; IBM Corporation, Somer, New York, USA).

## Results

### Production Parameters

Comparing selected production parameters collected at 35, 42, 45, or 50 days of rearing of healthy Iranian broiler flocks with data from the diseased farm clearly illustrated the decreased weight gain and increased mortality in the investigated flock (Table 1). Birds of the AGE affected flock were slaughtered at 50 days with a mean slaughter weight of 2,702 g. In comparison, healthy flocks reached equivalent weights already between 42 and 45 days of age. Furthermore, a higher total mortality rate was recorded for the AGE positive flock (plus 1.7%) compared to the average total mortality rate of the healthy broiler flocks at a corresponding slaughter age. In contrast, plant condemnation rate of birds from the affected flock was not different from the healthy flocks.

**Table 1:** Production parameters (body weight, total mortality, plant condemnation rate) of the investigated, positive flock in comparison to healthy broiler flocks slaughtered at 35, 42, 45, or 50 days of age.

	**Regional objectives^a^**	**Positive flock**
BW^b^ at 35 days (*n* = 14)	1868.9 ± 167.2 (1660 to 2202)	-
BW at 42 days (*n* = 22)	2522.3 ± 139.7 (2295 to 2785)	-
BW at 45 days (*n* = 22)	2739.1 ± 166.1 (2430 to 2969)	-
BW at 50 days (*n* = 10)	3036.3 ± 136.1 (2900 to 3350)	2702
Total mortality^c^ at 45 days (*n* = 22)	3.86 ± 1.35 (2 to 7)	5.8
Total mortality at 50 days (*n* = 10)	4.3 ± 1.2 (3 to 7.1)	6
Plant condemnation rate^d^ (%)	0.53 ± 0.41 (0.16 to 1.32)	0.52

a *Data presented as arithmetic mean ± standard deviation and minimum to maximum value in parentheses*.

b, c *BW (in g) and mortality (in %) of healthy broiler flocks raised concurrently and in the same region as affected broilers at 35, 42, 45, or 50 days of life, respectively (number of flocks assessed in parentheses)*.

d *Data recorded from 14 broiler flocks at the local processing plant during 3 consecutive working days*.

### Gross Pathology and Histology

**Figure 1:**
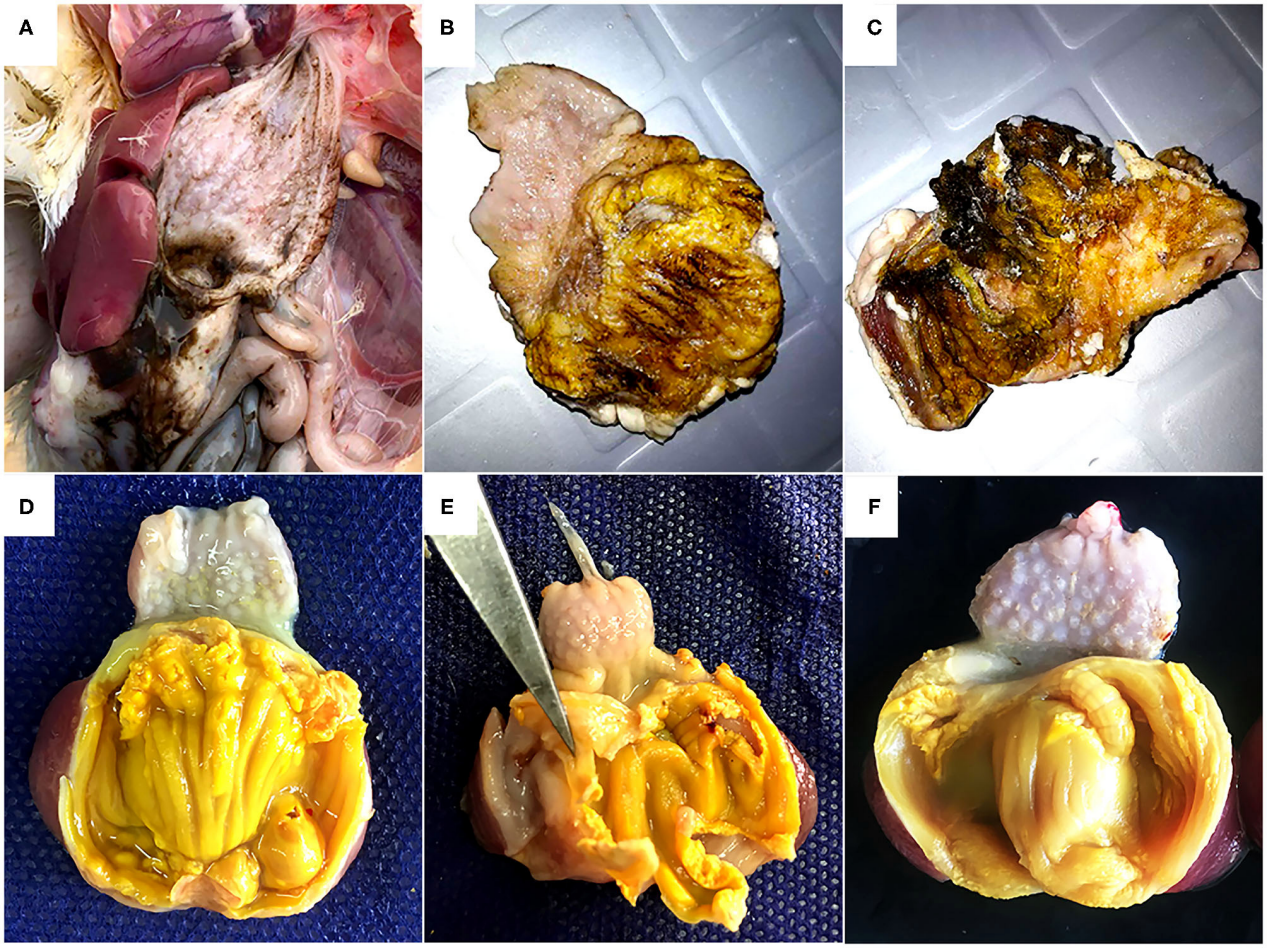
Gross pathology of gizzards from the field outbreak in 16-day old broilers **(A, B, C)** and commercial layers **(D, E, F)** experimentally infected on the first day of life with FAdV-1 (IRMGH019). **(A)** Black-brown, sanguineous fluid presenting at the cranial end of the gizzard. **(B)** Abrasion, discoloration and fragmentation of the koilin layer. **(C)** Disintegrated koilin layer with areas of erosion and inflammation of mucosal layer beneath. **(D)** Abrasion, dissociation, and perforation of the koilin layer in a chicken 7 days after inoculation with IRMGH019. **(E)** Detachment of koilin layer and inflammation of underlying mucosal layer in a chicken 10 days after inoculation with IRMGH019. **(F)** Mild abrasion and partial dissociation of the koilin layer in an in-pen contact bird 14 days after infection of the co-housed birds.

During necropsy, black fluids in crop, proventriculus, and gizzard coinciding with gizzard lesions of varying sizes characterized by koilin discoloration, abrasion and even disintegration were the most consistent findings (Figures 1A–C). No macroscopic lesions were noticed in other organs of the affected chickens. Histology revealed fragmentation and loss of the koilin layer. Mucosal epithelium showed necrosis and degeneration of the glandular epithelium cells. Mild to severe inflammatory cell infiltration was observed in gizzard mucosa, submucosa and to some extent in the muscular layer. H&E and ISH did not reveal intranuclear inclusion bodies.

### Virus Isolation, PCR and Sequencing

All of the gizzard samples exhibited characteristic CPE (rounded cells, detached from the flask) in cell culture. Sequencing and phylogenetic analysis of the hexon L1 PCR products obtained from cell culture supernatant confirmed the presence of FAdV-1 in all investigated samples. Furthermore, FAdV-1 long and short fiber specific PCRs yielded positive results for all samples.

**Figure 2:**
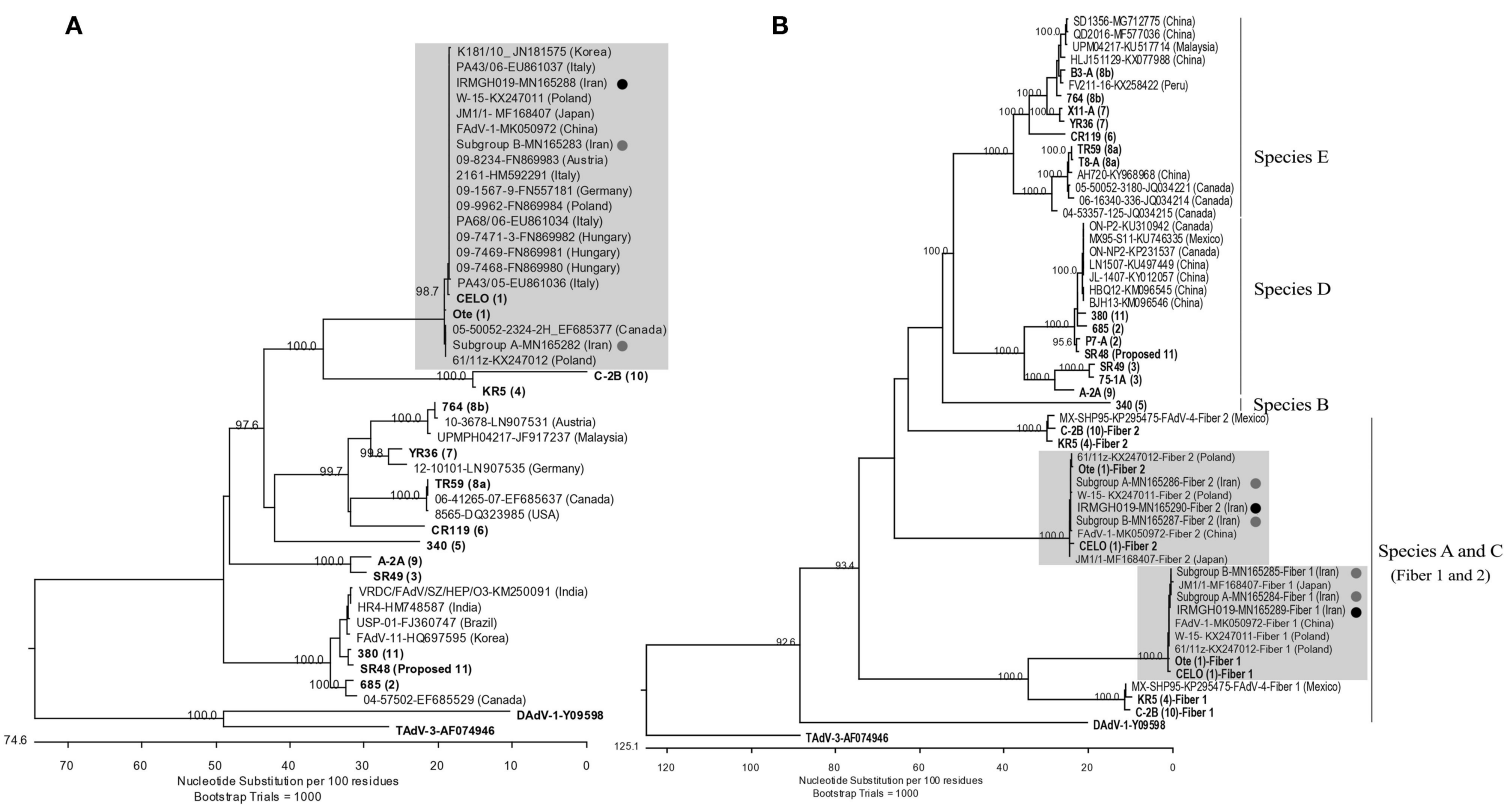
**(A)** Phylogenetic tree based on the partial hexon gene of avian adenoviruses, including the FAdV-1 isolates of this study and “subgroup A and B” isolates (17) (Designated with black/gray circles). Clustal W alignment was performed for a segment corresponding to nucleotides 18,632-19,268 of the complete CELO genome (GenBank accession no. U46993). **(B)** Phylogenetic tree based on the whole fiber gene of avian adenoviruses, including the FAdV-1 isolate of the actual study, subgroup A/B isolates, and FAdV available from GeneBank. For both trees, the reference isolates are in bold and the numbers in parentheses indicate serotypes. GenBank retrieved sequences are indicated by isolate's name, the accession number and country of origin. Representatives of the genera *Atadenovirus* (Duck adenovirus 1/DAdV-1) and *Siadenovirus* (Turkey adenovirus 3/TAdV-3) were used as outliers. Gray boxes indicate the FAdV-1 clusters.

Based on the hexon L1 gene region, all virus isolates obtained in this study were 100% identical. They were also identical to “pathogenic” European FAdV-1 strains documented by Marek et al. (21) as well as Asian AGE associated FAdV-1 strains from Japan (MF168407), Korea (12), and one of the FAdV-1 subcluster isolates from Iran (“subgroup B”) which we have reported recently (17). Other Iranian field isolates from our previous study (“subgroup A”) represented the most distant hexon with 98.9% identity. Furthermore, the isolates of this study possessed 99.7 and 99.2% hexon nucleotide identity to the reference sequences of Chicken Embryo Lethal Orphan (CELO) virus and Ote, respectively (Figure 2A).

Likewise, all field isolates of this study shared identical sequences in their long and short fiber genes. Furthermore, their nucleotide sequence identities to other FAdV-1 field strains available from GenBank ranged between 99.7–100% (long fiber) and 99.5–100% (short fiber). Both long and short fibers of the field isolates clustered more closely with reference strain Ote compared to CELO (99.8 vs. 99–99.1% nucleotide identity, respectively) (Figure 2B). The predicted fiber amino acid sequences revealed the same characteristics as the strains investigated by Marek et al. (21). Like in those strains, it was noted that all residue differences of the short fiber were concentrated in the C-distal knob domain (24), when compared with reference strain sequences, while residue differences of the long fiber were more dispersed.

### Pathogenicity of the Field Isolate in Day-Old Commercial Chicks

**Figure 3:**
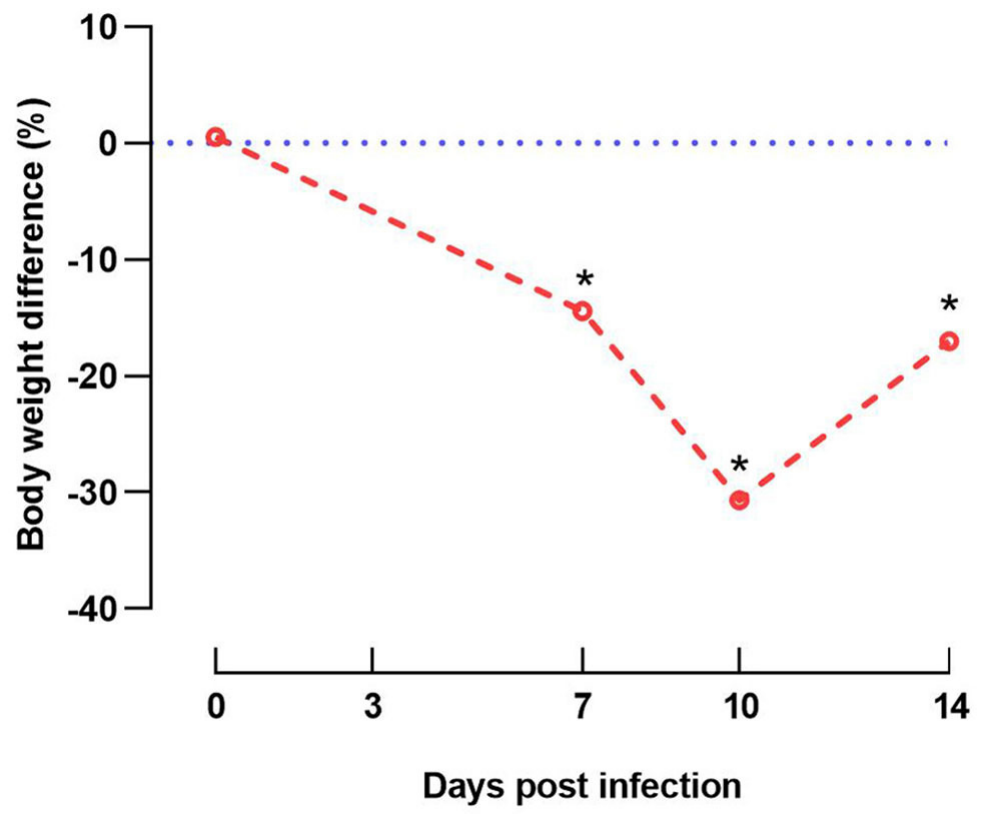
Graphical illustration of decreased weight gain. Difference of body weight (%) from experimentally infected and in-contact layer chickens (group 1) in comparison to respective negative control group at 0, 7, 10, and 14 days post infection. Data at 14 days post infection are based only on in-pen contact birds that survived until the end of the experiment. Asterisks indicate a significant difference from negative control group (*P* < 0.05).

Clinical signs including depression and anorexia were observed at 2 DPI in some of the inoculated chicks and continued until inoculated birds were necropsied (10 DPI). Mortality started at 5 DPI and persisted until 7 DPI; three, two, and one inoculated birds were found dead on 5, 6, and 7 DPI, respectively. No clinical signs were observed in in-pen contact chickens for the duration of the study. Compared with the uninfected control group, group 1 (inoculated and in-pen contact birds) showed significantly decreased (*P* < 0.05) weight gain from 7 DPI until the end of the experiment (Figure 3).

*Post-mortem* examinations of the dead and euthanized inoculated birds (at 5 to 10 DPI) revealed multiple erosions and extensive detachment of the koilin layer together with inflammation of the underlying mucosal layer in the gizzards (Figures 1D,E). Gross lesions appeared later in the in-pen contact birds with macroscopic lesions observed from 10 days post exposure onwards with less severity in comparison to the inoculated birds (Figure 1F) (Table 2). Presence of black-brown contents in crop, proventriculus and gizzard, as observed in the field, was not noticed in any of the birds.

**Table 2:** Pathological changes in the gizzard of commercial layer chicks experimentally infected with FAdV-1 (IRMGH019) at first day of life.

**Group**	**DPI**	**Macroscopic lesions of the koilin layer^a^**	**Histopathological changes^b^**	**Presence of inclusion bodies**
Inoculated	5	3/3 (2.3)	3/3	3/3
birds	6	2/2 (2.5)	2/2	2/2
	7	6/6 (2.6)	6/6	6/6
	10	5/5 (2.8)	5/5	4/5
In-pen	7	0/2 (0.0)	0/2	0/2
contact birds	10	2/2 (1.5)	2/2	2/2
	14	4/4 (1.6)	4/4	2/4

a *No. birds positive/no. birds examined (mean lesion scores in parentheses; no lesion = 0, mild lesions = 1, moderate lesions = 2 and severe lesions = 3)*.

b *Ventriculitis with necrosis and degeneration of glandular epithelium*.

**Figure 4:**
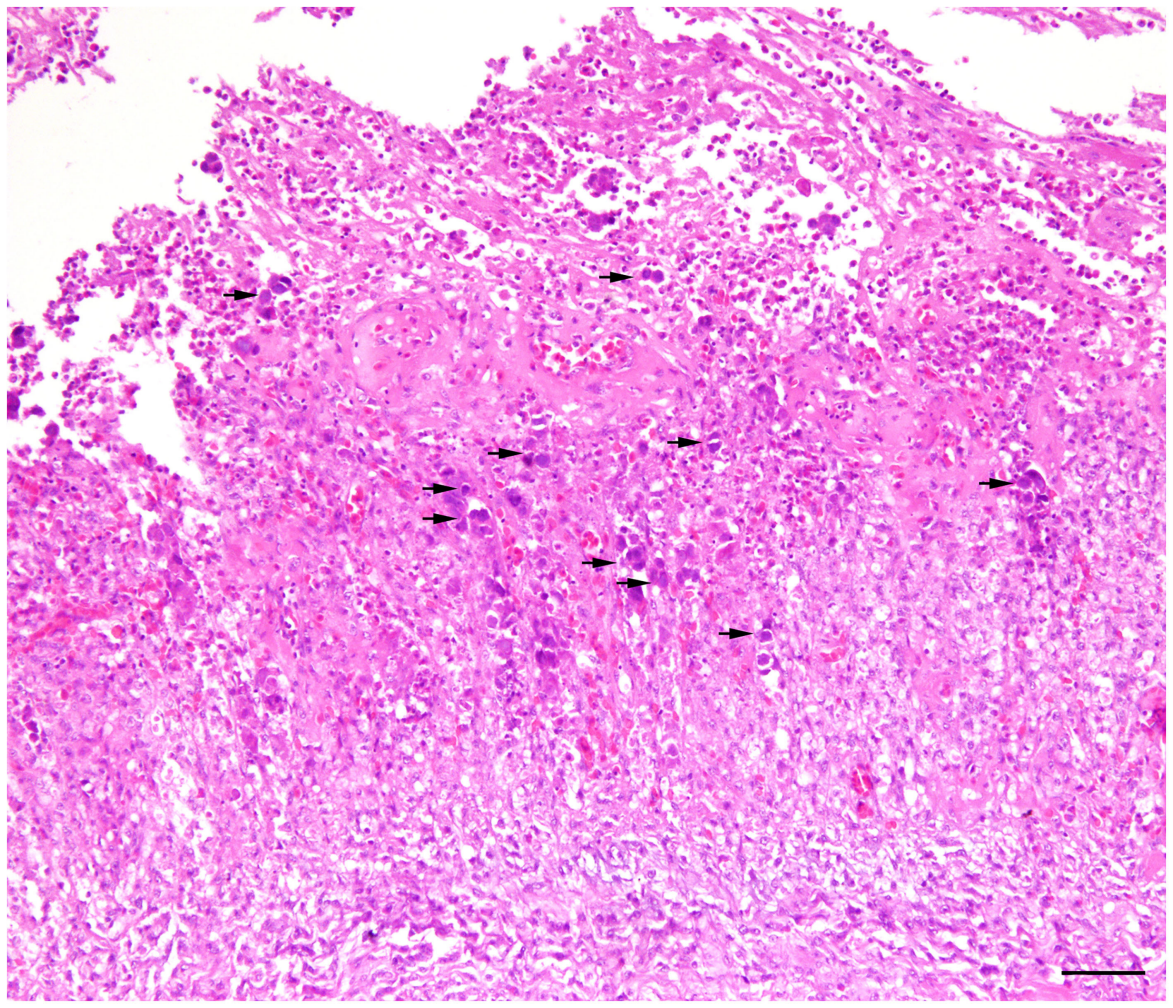
Histopathology of gizzard from a 7-day old commercial layer experimentally infected with FAdV-1 (IRMGH019) at first day of life. Extensive necrotizing ventriculitis characterized by loss and replacement of glandular epithelium by eosinophilic remnant and infiltration of the inflammatory cells. Large adenoviral inclusion bodies (arrows) are present inside of the necrotic areas. H & E. Bar = 50μm.

Histologically, frequent findings in affected gizzards (in both inoculated and in-pen contact birds) were compatible with observation from the field cases (necrosis and degeneration of the glandular epithelium accompanied by mild to severe infiltration of the inflammatory cells). However, intranuclear inclusion bodies were observed in affected gizzard epithelial cells (Figure 4). No clinical signs or gross lesions were found in the uninfected control birds.

## Discussion

In 1981, gizzard erosions were documented in Iranian broiler chickens although without recognizing a definite etiology at that time (25). Recently, AGE was documented at a slaughterhouse in Iran resulting in increased gizzard condemnations (17). The present study not only underlines previous findings, it also documents the first natural outbreak of AGE in Iran, in 16-day-old commercial broiler chickens. An initial macroscopic diagnosis was confirmed by molecular virology together with analysis of production data from the affected flock. The etiology was further substantiated by a subsequent experimental study reproducing the clinical signs and characteristic lesions of AGE with the FAdV-1 field isolate in day-old pullets.

While mortality has been recorded in the course of some AGE outbreaks, clinical observations in meat-type chickens are generally characterized by growth retardation with subsequently reduced harvest weights and/or condemnation of the gizzards at slaughterhouses (15). In the present case, increased mortality (0.16% per day) was the first clinical indication of AGE. Mortality continued concurrent with the detection of pathological changes in gizzards of affected birds for a period of 2 weeks resulting in a total loss of 6% which was 1.7% higher than the average rate recorded from healthy flocks in the region. A similar increase in mortality rates was observed in broilers during previous outbreaks of the disease (7, 9, 10). No mortality, but clinical signs of depression and uneven growth were noted by Domanska-Blicharz et al. (8) during an AGE outbreak in broilers in Poland. Similarly, reduced feed intake, subsequently reduced weight gains and flocks lacking uniformity were documented during natural outbreaks in Germany and Belgium (9–11). In our study, the growth retardation due to AGE became distinctively apparent at the end of the production cycle while comparing the average harvest weight of the affected flock with data from 10–22 healthy flocks raised contemporarily in the same region. AGE affected birds needed approximately one additional week in order to reach the defined target slaughter weight. Precise and continuous monitoring throughout the fattening period and/or retrospective analysis of slaughterhouse results are necessary in order to define actual production losses (9). Correspondingly, clinical signs (incl. depression and anorexia), increased mortality, and impaired weight gain, were also noticeable during the subsequent experimental study. The plant condemnation rate of the affected flock was low and similar to the rate of healthy flocks raised in parallel within the same timeframe, which was in agreement with previous findings in the course of the disease (9). Unfortunately, data on gizzard condemnation rate at slaughter was not available. However, the authors have previously reported a possible impact of AGE on flock productivity and marketability due to gizzard condemnation in Iran (17).

Gross examination of the affected chickens showed typical gizzard lesions corresponding with previously reported outbreaks (26). Likewise, microscopic findings were consistent with AGE, characterized by degradation of the koilin layer and necrosis of gizzard epithelial cells and infiltration with inflammatory cells. However, typical adenoviral inclusion bodies were not observed in the gizzard epithelial cells of the actual field samples. Such findings are in agreement with other studies reporting no or only low numbers of inclusion bodies in field samples from natural outbreaks of AGE (4, 14). Previous experimental AGE studies showed that the optimal time for histopathological inspection of intranuclear inclusion bodies was within the first week after infection (27). Induction of gizzard lesions in in-contact birds in the present *in vivo* study is in agreement with previous finding on efficient horizontal transmission of AGE (16). Possibly, a delayed and milder manifestation of gizzard lesion in these in-contact birds was due to exposure to lower titer of FAdV-1. In the field outbreak, mortality started in both houses of the farm at a similar age (16-day old). We speculate that the virus was introduced by vertical transmission infecting a fraction of birds within each barn. Consequently, a higher titer of virus would then be excreted in the feces and a large number of birds in both houses horizontally infected.

Based on reports of previous AGE outbreaks, the disease is commonly caused by infection with a pathogenic FAdV-1, which was confirmed by isolation of the virus from affected gizzard samples and by experimental reproduction of AGE in young broilers or SPF layer-type chickens infected orally or via nasal/ocular inoculation with field isolates (15). The isolation and identification of FAdV-1 from gizzard samples in the course of the present study and successful reproduction of AGE using the field isolate are in line with these reports. Virulence factors of FAdV-1 leading to the clinical manifestation of AGE are still under investigation. However, distinctive sequence variations were detected between pathogenic and non-pathogenic FAdV-1 strains in hexon and fiber genes (21, 28). The FAdV-1 isolated in the actual study shared 100% sequence identity in the hypervariable hexon L1 segment to European pathogenic strains (21) and “subgroup B” AGE-associated subcluster isolates previously defined by the authors (17). Comparing amino acid sequences of the long and short fiber genes of the FAdV-1 isolated in this study to apathogenic FAdV-1 reference strains CELO and Ote showed the same characteristics as reported by Marek et al. (21). However, as it was noted before, presence of these mutations in pathogenic strains as well as in either CELO or Ote, contests their role in pathogenicity. Although sequence conservation in all three investigated genes was relatively high (98.9–100%), the field isolate was more closely related to CELO reference strain based on hexon gene and showed more homology to Ote reference strain based on both long and short fibers. A concentration of polymorphisms was noted in the region of the short fiber which maps to the C-distal knob. Considering that the knob is the responsible domain for binding (29), and that particularly the FAdV-1 short fiber is critical for host-cell specific attachment (30), it may be hypothesized that this specific region of the short fiber is under increased selective pressure.

To the authors' knowledge, this is the first detailed description of a natural outbreak of AGE in 16-day-old commercial broilers from Iran. Present investigations document considerable economic losses due to increased mortality and significant growth retardation in the course of the FAdV-1-induced gizzard erosions both in the field and in an experimental setting. The study provides further insight in the epidemiology and far reaching consequences of the disease and argues for precise monitoring and diagnostics as well as efficacious control programs.

## Data Availability Statement

The nucleotide sequences for partial hexon, long, and short fiber genes obtained in this study have been submitted to GenBank under accession numbers MN165288, MN165289 and MN165290, respectively.

## Ethics Statement

The animal study was reviewed and approved by Institutional Committee on Animal Ethics, Shiraz University, Shiraz, Iran.

## Author Contributions

AM and SE-J directed farm investigation and sample collection, storage, and distribution. AM performed histopathology, virology, molecular investigations, and data analysis thereof, designed the experimental study and drafted the manuscript. MA and BA-H performed the experimental study, sample/data collection and related histopathological investigation. MH, BG, and AS critically reviewed the manuscript. All authors read and approved the final manuscript.

## Conflict of Interest

The authors declare that the research was conducted in the absence of any commercial or financial relationships that could be construed as a potential conflict of interest.
